# Hypovitaminosis D: A Disease Marker in Hospitalized Very Old Persons at Risk of Malnutrition

**DOI:** 10.3390/nu11010128

**Published:** 2019-01-09

**Authors:** Virginia Boccardi, Maria Lapenna, Lorenzo Gaggi, Francesco Maria Garaffa, Michele Francesco Croce, Marta Baroni, Sara Ercolani, Patrizia Mecocci, Carmelinda Ruggiero

**Affiliations:** Institute of Gerontology and Geriatrics, Santa Maria della Misericordia Hospital, Department of Medicine, University of Perugia, 06123 Perugia PG, Italy; marialapenna89@gmail.com (M.L.); lorenzo.gaggi.ger@gmail.com (L.G.); francescomaria.garaffa@studenti.unipg.it (F.M.G.); michelecroce@hotmail.it (M.F.C.); martabaroni@libero.it (M.B.); saraercolanisara@gmail.com (S.E.); patrizia.mecocci@unipg.it (P.M.); carmelinda.ruggiero@unipg.it (C.R.)

**Keywords:** biomarkers, geriatric assessment, hospital-related, multimorbidities, malnutrition, vitamin D

## Abstract

Background: Hypovitaminosis D is a frequent condition in elderly subjects. Vitamin D adequacy is best determined by measurement of the 25-hydroxyvitamin D-25(OH)D-concentration in the serum. An inverse association exists between 25(OH)D and cardiovascular, infectious, glucose metabolism, cognitive disorders, and all-cause mortality. Whether 25(OH)D is a marker of organ diseases is still under debate. We aimed to investigate whether comorbidities were associated with serum 25(OH)D levels in geriatric inpatients. Methods: This is a retrospective study, including 237 subjects consecutively admitted to an acute care geriatric unit, with available data of 25(OH)D serum concentrations. 25(OH)D serum levels were defined according to the following cutoffs: 50–30 ng/mL (125–75 nmol/L): optimal range; 30–20 ng/mL (75–50 nmol/L): insufficiency; 20–10 ng/mL (5–25 nmol/L): deficiency; and <10 ng/mL (<25 nmol/L): severe deficiency. Comorbidity was assessed using the Cumulative Illness Rating Scale-Geriatric (CIRS-G). Two summary measures were obtained, the Illness Severity Index (CIRS-SI) and the Comorbidity Index (CIRS-CI). Results: 177 (74.68%) women and 60 (25.32%) men with mean age of 85 ± 6 years old were enrolled. The majority of subjects (68.6%) were at risk of malnutrition. Overall, the burden of comorbidity was 1.87 ± 1.33 for CIRS-CI and 1.18 ± 0.40 for CIRS-SI. 25(OH)D serum concentrations were 10.58 ± 7.68 ng/mL, with 98.7% of subjects having vitamin D below 30 ng/mL and 56.6% with severe deficiency. An inverse correlation was found between 25(OH)D and both CIRS-SI (*r*: −0.312; *p* < 0.0001) and CIRS-CI (*r*: −0.306; *p* < 0.0001). Independent of multiple covariates an inverse association between both CIRS-SI (*p* < 0.0001) and CIRS-CI (*p* < 0.0001) and 25(OH)D was confirmed. Both CIRS-SI (*r* = 0.251, *p* < 0.0001) and CIRS-CI (*r* = 0.137, *p* = 0.016) were positively correlated with the length of hospital stay. An inverse correlation was confirmed between serum 25(OH)D concentrations and CRP (*r* = −0.142; *p* = 0.041). CRP, in turn, positively correlated with CIRS-SI (*r* = 0.209, *p* = 0.003) and CIRS-CI (*r* = 0.158, *p* = 0.023). Both CIRS-SI (*r* = 0.251, *p* < 0.0001) and CIRS-CI (*r* = 0.137, *p* = 0.016) were positively correlated with the length of hospital stay. Conclusions: In hospitalized very old subjects, a higher comorbidity burden is associated with lower 25(OH)D serum levels. Hypovitaminosis D was correlated with higher inflammatory status, which, together with the comorbidities burden, negatively influenced the length of hospital stay.

## 1. Introduction

Vitamin D and parathyroid hormone (PTH) are two major regulators of mineral metabolism. They play critical roles in the maintenance of calcium and phosphate homeostasis as well as the development and maintenance of bone health. Hypovitaminosis D is a highly prevalent condition among older adults, ranging in occurrence from 50% to 90%, depending on the definition used and the setting [1,2]. Vitamin D deficiency most commonly results from inadequate sunlight exposure but can also be caused by inadequate nutritional intake or disorders limiting its metabolism [2].

Vitamin D status is best determined by measurement of the 25-hydroxyvitamin D-25(OH)D-concentration in the serum. Several observational studies found that the concentration of 25(OH)D is inversely associated with all-cause mortality in the general population [3], as well as with the development and progression of several conditions, including cancer, cardiovascular diseases, glucose metabolism disorders, neurodegenerative diseases, infections, and autoimmune conditions [4,5,6].

In hospitalized adults, the greater the severity of chronic diseases the lower the 25(OH)D serum concentration, irrespective of the number of diseases [7], with a higher risk of clinical instability [8] and mortality [9,10]. Also, critical hospitalized patients with hypovitaminosis D experience an increased risk of complications from hospital-acquired infections [11] and a longer length of hospital stay, suggesting that hypovitaminosis D may be a marker of severity of comorbidity and length of hospital stay [12,13,14].

However, systematic reviews and metanalysis investigating the effects of vitamin D supplementation on disease occurrence and progression failed to show encouraging findings, leading to the hypothesis that serum 25(OH)D concentrations would essentially be a marker of illness status [15]. Ultimately, a randomized, double-blind trial revealed that the administration of high-dose vitamin D3 compared with placebo reduced hospital mortality among critically ill adult patients with severe vitamin D deficiency, without reducing the length of hospital stay [16]. Thus, other factors may have a more significant role in determining the length of hospital stay. The oldest critically ill patients usually have severe vitamin D deficiency [17] and suffer a higher burden of comorbidity, and therefore may benefit more from interventions. Thus, we aimed to investigate: (1) the association between serum 25(OH)D and comorbidities (2) the relationship between vitamin D levels at admission and length of hospital stay in very old subjects admitted to an acute geriatric care unit.

## 2. Materials and Methods

*Subjects and study design.* This is a retrospective study conducted among 860 older adults consecutively admitted to the geriatric acute care unit of Santa Maria della Misericordia Hospital (University Hospital of Perugia) from January 2017 to January 2018. Only subjects aged 65 years and more, with available measurement of serum 25(OH)D at admission, who were able to give written informed consent, were included. Vitamin D and PTH form a tightly controlled feedback cycle, with PTH being a major stimulator of vitamin D synthesis while vitamin D exerts negative feedback on PTH secretion. Thus, subjects with hyperparathyroidism, fragility fractures within three months, or ongoing oral vitamin D supplementation were ruled out. A final cohort of 237 patients was included in the study. Data on demographics, anthropometrics, physical examination, clinical and biochemical characteristics were gathered from the hospitalization chart. The ethical committee of the University Hospital of Perugia approved the study protocol.

*Analytical method.* Blood samples were collected in the morning after the participants had been fasting for at least eight hours. Intact parathyroid hormone (PTHi), albumin, calcium, phosphorous, magnesium, creatinine, and C-reactive protein levels (CRP) were determined in serum by routine laboratory methods (Roche Diagnostics, GmbH, Mannheim, Germany). Clearance creatinine has been calculated by the BIS-1 (Berlin Initiative Study) formula and expressed as mL/min/1.73 m^2^. Serum 25(OH)D concentrations were detected using the same routine test, for all subjects, at the hospital’s laboratory by chemiluminescence immunoassays (CLIA), according to standard protocol.

*Group definition.* Subjects were divided into four groups according to the serum concentration of 25(OH)D at admission. These are the thresholds used in the study [18]: optimal range (30–50 ng/mL), insufficiency (20–30 ng/mL), deficiency (10–20 ng/mL) and severe deficiency (<10 ng/mL). In addition, seasonality was determined considering fall (September, October, and November), winter (December, January, and February), spring (March, April, and May) and, summer (June, July, and August).

*Cognitive, functional, and nutritional assessment.* Cognitive performance was evaluated with the Mini Mental State Examination (MMSE) as a test of general cognition [19]. To avoid the underestimation of a self-rated level of functional capacity, an informant-based rating of functional status was carried out using the Basic Activities of Daily Living (BADL) [20] and the Instrumental Activities of Daily Living (IADL) scales [21]. BADL includes six activities: bathing, dressing, toileting, transferring, continence, and feeding. IADL includes eight activities: using the telephone, shopping, meal preparation, housekeeping, laundry, use of transportation, self-administration of drugs, and handling finances. Any dysfunction in the performances of these activities was recorded as dependence in the correspondent item. Because IADL items are often gender-specific, we used the version of the scale tested for male subjects that included only five items, with housekeeping, cooking, and doing laundry excluded. The BADL score ranges from 6 (total independence) to 0 (total dependence), and IADL from 8 (total independence) to 0 (total dependence) in women and from 5 (total independence) to 0 (total dependence) in men. The nutritional status was assessed by the administration of the Mini Nutritional Assessment (MNA) [22]. The MNA has been developed to assess malnutrition in elderly subjects and to select those who might benefit from early diagnosis and treatment. It is completed by a medical doctor and is comprised of 18 questions on (1) anthropometry, (2) dietary intake and habits, (3) general assessment, and (4) self-assessment. After completing the whole questionnaire, the total score (a maximum of 30 points) allows for grouping the nutritional status according to clearly defined boundaries: above 24 is defined as good status; 17‒23.5 means at risk of malnutrition; below 17 is defined as malnourished [22].

*Comorbidity.* Comorbidity was evaluated with the Cumulative Illness Rating Scale (CIRS) [23]. This rating scale consists of 14 items covering heart, hypertension, vascular and respiratory disorders, a combined eye‒ear‒nose‒throat item, upper and lower gastrointestinal systems, hepatobiliary system, kidney, genitourinary diseases, musculoskeletal diseases, endocrine/metabolic disorders, neurological system, and behavioral‒psychiatric disorders. Severity in each single item is rated according to the following algorithm: 1 = no, 2 = mild, 3 = moderate, 4 = severe, 5 = life-threatening. After the completion of CIRS by a medical doctor, two summary measures can be constructed: the illness Severity Index (CIRS-SI), which reflects the overall severity of diseases and the average rating of the 14 CIRS items, and the Comorbidity Index (CIRS-CI), computed by counting the number of items with a score ≥ 3 (moderate to severe pathology). As a result, the CIRS-CI can be considered the number of clinically relevant concomitant diseases.

*Statistical analyses.* The observed data were normally distributed (Shapiro‒Wilk W-test) and are presented as means ± standard deviation (SD). To assess differences among groups, unpaired *t*-test, ANOVA or Pearson’s Chi-squared (χ^2^) test were used, as appropriate. Also, the Manthel‒Hanzel test for trends was tested among groups. Simple and partial (controlling for age and gender) correlations were used to test relations between serum 25 (OH)D concentrations, comorbidity indices, CRP, and length of hospital stay (LOS). The independent effect of comorbidities burden on 25(OH)D concentrations (dependent variable) was tested by a linear regression controlling by multiple covariates, including age, gender, season, albumin, BADL, IADL, and renal function. In this model gender is indicated as M = 1 and F = 0, while season is winter = 1, spring = 2, summer = 3, autumn = 4. All *p* values are two-tailed, and the level of significance was set at *p* ≤ 0.05. Statistical analyses were performed using the SPSS 20 software package (SPSS, Inc., Chicago, IL, USA).

## 3. Results

### 3.1. Sample Characteristics

Table 1 shows the baseline characteristics of the whole cohort and is stratified by gender. Participants were more likely women (177; 74.68%), with a mean age of 86.5 ± 6.2 years and with a high prevalence of comorbidity and polypharmacy. Comorbidity indices were indicative of a high burden of disease interfering with normal activity and requiring intensive care at admission. Participants were taking a mean of 6.51 drugs per day, with a rate of polypharmacy defined as >5 drugs and affecting 66.7% of participants. Overall, 13% of the participants had cognitive impairment, defined as MMSE score corrected by age and education lower than 23. From a functional perspective, 131 subjects (55.2%) had BADL ≤ 3, 140 women (79%) had IADL ≤ 4, and seven men (11.6%) had IADL ≤2, indicative of severe disability. In the whole cohort, 68.6% of subjects had an MNA score ≤ 23.5, indicative of a malnutrition risk status or state of being malnourished. The mean 25(OH)D serum concentration was indicative of deficiency (10.58 ± 7.68 ng/mL), with 56% of patients affected by severe deficiency. They had mean levels of PTHi indicative of secondary hyperparathyroidism. No gender interaction was found between the serum concentration of vitamin D and PTHi. No difference was found in the length of hospital stay between genders. In the whole population an inverse correlation between 25(OH)D serum concentration and PTHi (*r* = −0.280, *p* < 0.0001), controlling for age and gender, was found. No correlation was found between 25(OH)D and calcium (*p* = 0.887), phosphorus (*p* = 0.110), and magnesium (*p* = 0.099).

### 3.2. Sample Characteristics Stratified by Vitamin D Status

Almost the entire sample (98.7%) had 25(OH)D insufficiency, with one in two and one in three participants affected by a severe deficiency (56.6%) and deficiency (29.9%), respectively. Only three patients (1.3%) were in the optimal range (Table 2). In the severe deficiency groups, subjects were older, were more dependent in IADL, more likely malnourished, and had a high level of PTHi. Participants with severe deficiency of 25(OH)D had a significantly higher comorbidity index and comorbidity severity index as compared with other groups, with a statistically significant trend across groups (*p* < 0.0001). No difference was found in the length of hospital stay among vitamin D status groups. Table 3 shows the rate of vitamin D status in different seasons in the whole population sample. No significant difference was found in vitamin D status among seasons.

### 3.3. Vitamin D, Comorbidities Burden, CRP, and Length of Hospital Stay

**Figure 1:**
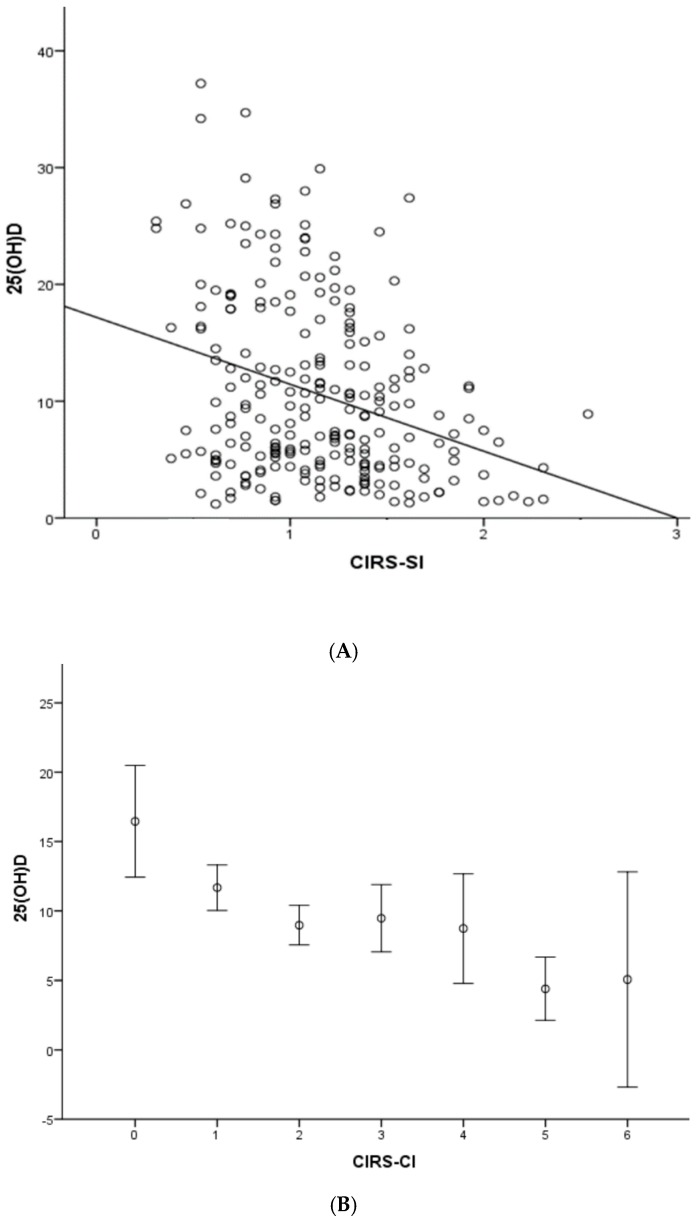
Simple correlation analysis between serum 25 (OH)D concentrations and CIRS-SI (**A**) and CIRS-CI (**B**), in the whole cohort (*n* = 237). 25(OH)D: 25-hydroxyvitamin D expressed in ng/mL; CIRS-SI: Cumulative Illness Rating Scale-Severity index; CIRS-CI: Cumulative Illness Rating Scale-Comorbidity index. (**A**) Simple correlation between 25(OH)D concentration and CIRS-SI scores (*r*: −0.312; *p* < 0.0001); (**B**) simple correlation between 25(OH)D concentration and CIRS-CI (*r*: −0.306; *p* < 0.0001). *r* = Pearson’s correlation coefficient; *p* = statistical significance of Pearson’s correlation coefficient.

An inverse correlation was found between serum 25 (OH)D concentrations and comorbidity indices CIRS-SI (*r*: −0.312; *p* < 0.0001) and CIRS-CI (*r*: −0.306; *p* < 0.0001) in the entire sample (Figure 1A,B), even after adjustment for age and gender (*r*: −0.281; *p* < 0.0001 and *r*: −0.286; *p* < 0.0001, respectively). The independent effect of comorbidities burden on 25(OH)D levels was tested by a linear regression analysis controlling by multiple covariates. CIRS-SI (Table 4, Model 1) and CIRS-CI (Table 4, Model 2) remained inversely associated with 25(OH)D concentrations independent of age, gender, season, albumin, BADL, IADL, and renal function.

An inverse correlation was confirmed between serum 25(OH)D concentrations and CRP (*r* = −0.142; *p* = 0.041). As expected, CRP was also positively correlated with CIRS-SI (*r* = 0.209, *p* = 0.003) and CIRS-CI (*r* = 0.158, *p* = 0.023), but not with the length of hospital stay. Both CIRS-SI (*r* = 0.251, *p* < 0.0001) and CIRS-CI (*r* = 0.137, *p* = 0.016) were positively correlated with the length of hospital stay, while no significant correlation was found between 25(OH)D concentrations and length of hospital stay (*r* = −0.006, *p* = 0.932). In a final model with length of stay as the dependent variable and Vitamin D, CIRS-SI, and CRP as covariates, only CIRS-SI resulted significantly associated (data not shown).

## 4. Discussion

Our results show that (1) the majority of hospitalized older persons have deficient levels of vitamin D, irrespective of season; (2) indices of comorbidity burden are negatively associated with 25(OH)D serum concentrations independent of multiple confounding factors; (3) 25(OH)D negatively correlates with serum CRP levels; and (4) the comorbidities burden is correlated with higher CRP levels and longer length of hospital stay.

Vitamin D deficiency is increasingly recognized as a common problem among older persons, including medical [24], surgical [25], and critically ill hospitalized patients [26]. Consistent with previous findings, 98.7% of our oldest-old hospitalized population had hypovitaminosis D [24,25,26].

In this cohort, subjects with severe deficiency in 25(OH)D concentration have a higher comorbidity burden, in term of both severity and complexity (CIRS-SI and CIRS-CI), as compared with those in the optimal range group As expected, a person affected by multiple diseases and with poor health status may curtail outdoor activities, may undergo changes in diet and lifestyle with low dietary intake of vitamin D, low sunlight exposure, or may require drugs affecting vitamin D absorption or metabolism. Thus, low vitamin D concentrations may act as a surrogate of poor health rather than a marker of impaired cellular functions. On the other hand, it is possible to hypothesize that the comorbidity per se can directly exert an influence on the serum concentrations of vitamin D, as supported by the inverse association between 25(OH)D and comorbidity indices independent of multiple covariates including age, gender, season, albumin level (as an indicator of nutritional status), and functional status.

Vitamin D plays a critical role in the functioning of the immune system [27]. Interestingly, a direct and inverse correlation between 25(OH)D concentrations and CRP, a sensitive biomarker for inflammation, has been found in some studies [28]. We found a significant inverse correlation between 25(OH)D and serum levels of CRP. Interestingly, CRP levels reflect the severity of response to injury in an acute care setting [29] and may be a determinant of clinical outcome, including the length of hospital stay. Here we found a correlation between CRP levels and comorbidities burden, which in turn is associated with the length of stay.

Many previous studies have proposed vitamin D as a biomarker of a longer length of stay in acute care units [30,31]. Hypovitaminosis D doubled the risk of being hospitalized for more than 14 days in a geriatric acute care unit [30] and, in another study, the combination of hypovitaminosis D, male gender, and delirium predicted a 4.8-fold higher risk of longer LOS among geriatric inpatients [31]. Another study showed that older patients with serum 25(OH)D ≤ 50 nmol/L at the time of admission to the hospital had a LOS extended by approximately three days compared to those with 25(OH)D > 50 nmol/L [14]. Additionally, they reported the first evidence of a linear association between 25(OH)D concentrations and LOS, suggesting that any increase in 25(OH)D was associated with a decrease in LOS, irrespective of the initial vitamin D status [14]. We did not find a difference in the length of hospital stay among vitamin D status groups. A causal relationship between vitamin D levels and this specific outcome cannot be directly established considering that other variables could potentially influence the LOS: functional status score, illness severity, cognitive score, poor nutrition, comorbidity score, diagnosis or presenting illness, polypharmacy, age, and gender. A prospective multicenter study that included 250 intensive care unit (ICU) patients shows that vitamin D insufficiency is common in critically ill patients (69%) and vitamin D-deficient patients had more severe diseases, according to the Acute Physiology and Chronic Health Disease Classification System II score, but this is not an independent risk factor for longer ICU stay or mortality [13]. Accordingly, we found that a higher comorbidities burden is associated with lower 25(OH)D serum concentrations independent of multiple covariates. Moreover, we found that both CIRS-SI and CIRS-CI indices significantly correlated with CRP as well as the length of hospital stay. These results strongly support the importance of the determination of 25 (OH)D as a potential measure of the patient’s comorbidity as well as vulnerability during hospitalization.

The current Italian guidelines state that measurement of serum 25(OH)D concentrations, whose cost is considerable, is not always justified from a health cost standpoint, especially in the elderly subject, as hypovitaminosis D is highly prevalent in this population. Neither routine nor screening assessments of serum 25(OH)D are recommended; they should be limited to doubtful cases or in the presence of comorbidities that increase the risk of severe hypercalcemia (e.g., granulomatosis) [17]. The National Osteoporosis Society (NOS) recommends the measurement of serum 25(OH)D to estimate vitamin D status only in the following clinical scenarios: bone diseases that may be improved with vitamin D treatment; bone diseases, prior to specific treatment where correcting vitamin D deficiency is appropriate; and musculoskeletal symptoms that could be attributed to vitamin D deficiency. The guidelines also state that routine vitamin D testing is unnecessary where vitamin D supplementation with an oral antiresorptive treatment is already planned.

It is challenging to clarify whether vitamin D deficiency is the cause or only the consequence of various chronic diseases. However, with this study, we provide evidence that vitamin D status may be used as a biomarker of comorbidities burden and indirectly as a determinant of clinical outcome, including LOS. Whether screening and supplementation of vitamin D deficiency can improve clinical outcomes and in particular LOS remains unclear. Variations in patient responses to acute and critical illness may depend on the degree of vitamin D insufficiency. Future studies are needed to determine how this marker can change over time and affect clinical outcomes.

## Figures and Tables

**Table 1:** Baseline population sample characteristics (*n* = 237).

	Total Sample *N* = 237	Men *N* = 60	Women *N* = 177	*p*
Age, y	86.5 ± 6.2	86.12 ± 6.45	87.18 ± 6.22	0.258
Drugs, n	6.51 ± 3.20	7.43 ± 3.08	6.19 ± 3.19	0.013
CIRS-SI	1.87 ± 1.33	2.15 ± 1.43	1.78 ± 1.28	0.062
CIRS-CI	1.18 ± 0.40	1.27 ± 0.41	1.16 ± 0.40	0.069
MMSE adjusted	22.8 ± 4.7	23.6 ± 4.5	22.8 ± 4.4	0.532
MMSE ≤ 23 (*n*, %)	31 (13.1)	4 (6.6)	27 (15.2)	0.063
BADL	2.76 ± 2.16	2.82 ± 2.39	2.73 ± 2.09	0.793
IADL	2.14 ± 2.70	2.23 ± 2.60	2.10 ± 2.66	0.756
MNA	21.08 ± 3.71	21.36 ± 3.50	21.04 ± 3.76	0.660
25(OH)D (ng/mL)	10.58 ± 7.68	9.53 ± 7.13	10.94 ± 7.83	0.218
PTHi (pg/mL)	112.9 ± 112.9	101.16 ± 85.38	116.00 ± 121.31	0.556
Albumin	3.26 ± 1.15	3.22 ± 1.01	3.28 ± 1.20	0.733
Calcium, corrected (mg/dL)	8.55 ± 2.47	8.68 ± 2.08	8.51 ± 2.59	0.651
Phosphorus (mg/dL)	2.70 ± 1.85	2.95 ± 1.91	2.61 ± 1.83	0.218
Magnesium (mg/dL)	1.20 ± 1.02	1.21 ± 1.03	1.19 ± 1.02	0.884
CRP (mg/L)	5.02 ± 6.07	6.15 ± 7.10	4.64 ± 5.66	0.120
Clearance creatinine (BIS1)	41.3 ± 19.5	42.23 ± 22.98	39.74 ± 18.00	0.027
Length of hospital stay (days)	12.33 ± 9.22	12.10 ± 9.28	10.01 ± 9.08	0.510

Unless otherwise noted, data are presented as means ± SD. CIRS-SI: Cumulative Illness Rating Scale-Severity index; CIRS-CI: Cumulative Illness Rating Scale-Comorbidity index; MMSE: Mini Mental State Examination (corrected by age and education); BADL: Basic Activities of daily living; IADL: Instrumental activities of daily living; MNA: Mini Nutritional Assessment; 25(OH)D: 25-hydroxyvitamin D; PTHi: Intact Parathyroid Hormone; CRP: C-reactive protein; Clearance creatinine calculated by BIS-1 (Berlin Initiative Study) formula and expressed as ml/min/1.73 m^2^.

**Table 2:** Clinical characteristics of patients stratified by vitamin D status.

	Severe Deficiency (<10 ng/mL)	Deficiency (10–20 ng/mL)	Insufficiency (20–30 ng/mL)	Optimal Range (30–50 ng/mL)	*p*
N (%)	134 (56.6)	71 (29.9)	29(13.2)	3(1.3)	
Age, y	87.8 ± 6.5	85.6 ± 6.4	85.7 ± 4.1	85.3 ± 2.0	0.057
Gender M/F, *n*	34/100	21/50	4/25	1/2	0.420
Gender M/F, %	24.4/74.6	29.5/70.5	13.8/86.2	87/12	0.420
Drugs, n	6.21 ± 2.97	7.25 ± 3.33	6.31 ± 3.87	4.50 ± 0.70	0.148
CIRS-SI	1.27 ± 0.43	1.14 ± 0.34	0.97 ± 0.32	0.71 ± 0.07	<0.0001
CIRS-CI	2.21 ± 1.42	1.56 ± 0.95	1.28 ± 1.25	0.0 ± 0.0	<0.0001
MMSE adjusted	22.40 ± 5.20	23.57 ± 4.05	23.23 ± 3.08	25.75 ± 2.21	0.465
BADL	2.40 ± 2.10	3.19 ± 2.93	3.26 ± 2.14	3.00 ± 2.64	0.056
IADL	1.61 ± 2.35	2.90 ± 2.93	2.48 ± 2.68	3.33 ± 3.51	0.009
MNA	20.36 ± 3.65	22.73 ± 2.87	19.95 ± 4.54	23.67 ± 0.57	0.001
PTHi (pg/mL)	136.7 ± 125.3	95.5 ± 101.4	61.9 ± 39.1	34.0 ± 8.1	0.012
Albumin (g/dL)	3.19 ± 1.16	3.35 ± 1.16	3.32 ± 1.16	3.5 ± 0.36	0.757
Calcium, corrected (mg/dL)	8.45 ± 2.44	8.47 ± 2.74	9.13 ± 1.99	9.42 ± 0.25	0.525
Phosphorus (mg/dL)	2.53 ± 1.99	2.76 ± 1.64	3.22 ± 1.70	3.73 ± 0.92	0.226
Magnesium (mg/dL)	1.08 ± 1.04	1.30 ± 0.99	1.46 ± 0.94	1.36 ± 1.18	0.205
CRP (mg/L)	5.51 ± 6.18	4.30 ± 5.98	4.70 ± 5.97	2.20 ± 2.40	0.546
Clearance creatinine (BIS1)	41.29 ± 18.75	42.65 ± 21.82	40.23 ± 18.13	27.50 ± 5.21	0.594
Length of hospital stay (days)	11.83 ± 8.14	13.00 ± 10.87	13.06 ± 9.81	12.00 ± 9.84	0.816

Unless otherwise noted, data are presented as means ± SD. CIRS-SI: Cumulative Illness Rating Scale-Severity index; CIRS-CI: Cumulative Illness Rating Scale-Comorbidity index; MMSE: Mini Mental State Examination (corrected by age and education); BADL: Basic Activities of daily living; IADL: Instrumental activities of daily living; MNA: Mini Nutritional Assessment; PTHi: Intact Parathyroid Hormone; CRP: C-reactive protein; Clearance creatinine calculated by BIS-1 (Berlin Initiative Study) formula and expressed as mL/min/1.73 m^2^.

**Table 3:** The rate of overall vitamin D status in different seasons in the whole cohort. *Χ^2^* = 10.055, *p* = 0.346.

Season	Severe Deficiency (<10 ng/mL)	Deficiency (10–20 ng/mL)	Insufficiency (20–30 ng/mL)	Optimal Range (30–50 ng/mL)
Fall *n* (%)	39 (56.5)	18 (26.1)	10(14.5)	2(2.9)
Winter *n* (%)	16 (44.4)	13 (36.1)	6(16.7)	1(2.8)
Spring *n* (%)	42 (65.6)	15 (23.4)	7(10.9)	0(0)
Summer *n* (%)	37 (54.4)	25 (36.8)	6(8.8)	0(0)

**Table 4:** Linear regression analyses to assess whether comorbidities burden is associated with 25(OH)D concentrations controlling for multiple confounding factors (*n* = 237).

**Model 1**	**B**	***p***
Age	−0.097	0.292
Gender	−1.083	0.363
Season	−0.583	0.181
Albumin	0.446	0.317
BADL	−0.047	0.900
IADL	0.034	0.926
BIS1	0.083	0.793
CIRS-SI	−6.322	<0.0001
**Model 2**	**B**	***p***
Age	−0.103	0.266
Gender	−1.386	0.242
Season	−0.726	0.101
Albumin	0.335	0.452
BADL	0.031	0.934
IADL	0.178	0.573
BIS1	0.000	0.995
CIRS-CI	−1.854	<0.0001

Gender indicated as M = 1 and F = 0; Season indicated as winter = 1, spring = 2, summer = 3, autumn = 4; BADL: Activity of daily living; IADL: instrumental activity of daily living; BIS1 Clearance creatinine calculated by BIS-1 (Berlin Initiative Study) formula and expressed as mL/min/1.73 m^2^; CIRS-SI: Cumulative Illness Rating Scale-Severity index; CIRS-CI: Cumulative Illness Rating Scale-Comorbidity index.
